# Combined R2R3–MYB transcription factor mutants reveal the regulatory structure of the *Arabidopsis thaliana* flavonoid biosynthesis pathway

**DOI:** 10.1007/s00425-026-04938-8

**Published:** 2026-02-06

**Authors:** Lennart Malte Sielmann, Timo Denecke, Bernd Weisshaar, Ralf Stracke

**Affiliations:** Genetics & Genomics of Plants, Faculty of Biology, Center for Biotechnology (CeBiTec), Bielefeld University, Sequenz 1, 33615 Bielefeld, Germany

**Keywords:** Anthocyanins, Flavonols, Multiple mutants, Proanthocyanidins, Transcription control

## Abstract

**Main conclusion:**

Comparative metabolic profiling of new genetic multiple flavonoid *r2r3-myb* mutants show that different types of R2R3–MYBs activate the early flavonoid biosynthesis genes. A revised model for flavonoid biosynthesis in *Arabidopsis thaliana* is proposed that integrates the regulatory roles of these R2R3–MYBs across early and late biosynthetic steps.

**Abstract:**

Flavonoids are a large group of specialized plant metabolites. Their biosynthesis is mainly transcriptionally regulated by a sophisticated network of different transcription factors from various families, with R2R3–MYB factors being the main determinant of specific flavonoid class formation. The early biosynthetic steps, leading to the formation of non-visible flavonoids, have been proposed to be regulated by three R2R3–MYBs, PRODUCTION OF FLAVONOL GYLCOSIDE1-3 (PFG1-3), while the later biosynthetic steps leading to the formation of visible anthocyanin and proanthocyanidin pigments are reported to be regulated by four R2R3–MYBs, PRODUCTION OF ANTHOCYANIN PIGMENT1-4 (PAP1-4) and TRANSPARENT TESTA2 (TT2), respectively. Several studies have indicated that this model for the transcriptional regulation of flavonoid biosynthesis may be incomplete. To address this issue, especially regarding the regulation of the early biosynthesis genes by PAP1-4 and TT2, we generated several multiple *r2r3-myb* mutant lines. We characterized the *pfg1-3*, *pfg1-3 tt2* and *pfg1-3 pap1-4* mutants and did comparative metabolite profiling. This revealed that only the *pfg1-3 tt2* mutant was deficient in proanthocyanidins and only the *pfg1-3 pap1-4* mutant was deficient in anthocyanins. We demonstrate that PAP and TT2 R2R3–MYBs are also capable of activating the early biosynthesis genes required for dihydroflavonol formation. Our results provide evidence that the traditional view of distinct branch-specific R2R3–MYB regulators in flavonoid biosynthesis is overly simplistic. We, therefore, propose a revised model for the transcriptional regulation of flavonoid biosynthesis.

**Supplementary Information:**

The online version contains supplementary material available at 10.1007/s00425-026-04938-8.

## Introduction

Flavonoid biosynthesis is one of the most-well-studied pathways of plant specialized metabolism. Flavonoids are a large group of phenolic compounds with an estimated number of over 9,000 different derivatives (Ferrer et al. 2008). They all share a common structure: a C6–C3–C6 skeleton, consisting of two aromatic C6 rings linked by a heterocyclic C3 ring. Modification of this core skeleton leads to diversification and production of a wide variety of different flavonoids, which can be sub-classified into several families, including flavonol, flavone, flavanone, flavan-3-ol, isoflavone and anthocyanin, each exhibiting distinct structural characteristics and biological functions (Wen et al. 2020). Three different main flavonoid subgroups are found in wild-type *Arabidopsis thaliana* (*A. thaliana*), the premier model plant for post-genomic biology: flavonols, anthocyanins and proanthocyanidins (PAs). Flavonols are regularly glycosylated and represent a combination of different aglycone types (such as kaempferol, quercetin, and isorhamnetin) linked to different sugar moieties (glucose, rhamnose and arabinose) in various combinations (mono-, di- or tri-). Flavonols have been implicated in seed longevity, plant growth regulation and UV protection, among other functions (Li et al. 1993; Gayomba et al. 2017; Ninoles et al. 2023). The visible anthocyanin pigments, which have been used as a stress marker in many studies, impact pollination and seed dispersal (Tanaka et al. 2008), UV protection, and have antioxidant activity (Gould 2004). PAs, or condensed tannins, are formed in *A. thaliana* from epicatechin and specifically accumulate in the seed coat as the seed maturates, conferring a brown color. They have been shown to act as deterrents, modulating the defense against pathogens and herbivores (Dixon et al. 2005), to exhibit antioxidant activity, and can play a structural role in cell wall stability (Lepikson-Neto et al. 2014; Demonsais et al. 2020a, 2020b). In addition to their roles in plants, flavonoids are studied for their potential health benefits in humans, including anti-inflammatory and antioxidant effects, although more research is needed to fully understand these effects (Durazzo et al. 2019).

Flavonoid scaffolds are synthesized in the central flavonoid biosynthetic pathway, also known as the early steps of flavonoid biosynthesis, from building blocks of the central phenylpropanoid pathways (Hahlbrock and Scheel 1989). p-Coumaroyl-CoA (from the phenylpropanoid pathway) is condensed with three malonyl-CoA molecules (from the polyketide pathway) by the action of chalcone synthase (CHS), forming naringenin chalcone. CHS is, therefore, defined as the key enzyme in flavonoid biosynthesis, as the formation of all flavonoid classes rely on its functionality. Naringenin chalcone is then converted into dihydroflavonols in a series of enzymatic steps, involving the enzymes chalcone isomerase (CHI), flavanone 3-hydroxylase (F3H) and flavonoid 3’-hydroxylase (F3’H) (Ferrer et al. 1999; Jez et al. 2000; Owens et al. 2008b).

The dihydroflavonols represent a branching point of flavonoid biosynthesis in *A. thaliana*. The enzyme flavonol synthase (FLS) converts dihydroflavonols into flavonols, which are then stabilized into flavonol glycosides by glycosyl transferases (Winkel-Shirley 2001; Owens et al. 2008a; Saito et al. 2013; Zhu et al. 2025). Alternatively, the enzyme dihydroflavonol reductase (DFR) can reduce the dihydroflavonols to leucoanthocyanidins (Davies et al. 2003), which can be converted to anthocyanidins by the enzyme anthocyanidin synthase (ANS) (Wilmouth et al. 2002).

The formation of anthocyanidins is the next branching point in flavonoid biosynthesis, as anthocyanidins are the precursors of both anthocyanins and PAs. Glycosylation and other modifications of the colored anthocyanidins produce the more water soluble and stable anthocyanins, while alternatively, the enzyme anthocyanidin reductase (ANR) can catalyze the conversion to the flavan-3-ol epicatechin (Xie et al. 2003; Yonekura-Sakakibara et al. 2012), which is the basic compound of PAs, thatreduced transcript levels in the are formed by polymerization to form oligomers and polymers (Debeaujon et al. 2003).

The flavonoid biosynthesis in *A. thaliana* can, therefore, be broken down into three branches, leading to the formation of flavonols, anthocyanins, and PAs. Dihydroflavonols and anthocyanidins are found at the branching points.

The activity of the enzyme encoding "structural" genes of flavonoid biosynthesis in *A. thaliana* are controlled by a sophisticated regulatory network, involving members of numerous transcription factor (TF) families, including WIP-type, MADS-box, bZIP, WD40 and WRKY TFs (Johnson et al. 2002; Nesi et al. 2002; Sagasser et al. 2002; Stracke et al. 2010a). The TF families of MYB and bHLH have been shown to be of crucial importance. The *A. thaliana* R2R3–MYB TFs PRODUCTION OF FLAVONOL GLYCOSIDE1-3 (PFG1/MYB12, PFG2/MYB11, and PFG3/MYB111), which form the subgroup 7 (SG7) of the R2R3–MYB family (Stracke et al. 2001), have been shown to activate the early biosynthesis genes *(EBGs)* and the flavonol branch (Stracke et al. 2007) in the plant body, except of pollen and anthers (Stracke et al. 2010b). Here, the SG19 MYBs, MYB21, MYB24, and MYB57 and MYB99, were shown to regulate flavonol accumulation (Battat et al. 2019; Zhang et al. 2021).

The late biosynthetic genes *(LBGs)* in the anthocyanin and proanthocyanidin branches were reported to be regulated by the SG6 R2R3–MYBs, PRODUCTION OF ANTHOCYANIN PIGMENT1-4 (PAP1/MYB75, PAP2/MYB90, PAP3/MYB113, PAP4/MYB114) and the SG5 R2R3–MYB, TRANSPARENT TESTA2 (TT2/MYB123), respectively (Borevitz et al. 2000; Nesi et al. 2001; Gonzalez et al. 2008; Xu et al. 2015). While the TFs PFG1-3 act independently of any known co-factors (Stracke et al. 2007), the TFs PAP1/PAP2 and TT2 form ternary complexes involving group IIIf basic helix–loop–helix (bHLH) proteins and the WD40 repeat protein (WDR) TRANSPARENT TESTA GLABRA1 (TTG1) (Baudry et al. 2004; Thévenin et al. 2012). The bHLH protein interacting R2R3–MYBs contain a conserved amino acid signature ([DE]Lx2[RK]x3Lx6Lx3R) as the structural basis for interaction between MYB and bHLH (Zimmermann et al. 2004). These ternary complexes are known as MYB–bHLH–WDR complexes (MBW-complexes). Several MBW-complexes have been reported in *A. thaliana*, including the complex of PAP1–EGL3–TTG1 regulating the anthocyanin biosynthesis branch and TT2–TT8–TTG1 regulating the PA biosynthesis branch (Gonzalez et al. 2008; Xu et al. 2013). Although both, R2R3–MYB and bHLH TFs, have been shown to be capable of DNA binding; however, the target gene specificity is thought to rely primarily on the R2R3–MYB and its DNA binding domain (Heppel et al. 2013).

Several loss-of-function mutants of flavonoid biosynthesis regulators in *A. thaliana* have been isolated and characterized in previous studies. The *tt2* mutant, as described by Nesi et al. (2001), has been found to be deficient in PAs, but to still accumulate flavonols and anthocyanins. The *pfg1-3* loss-of-function mutant, as described by Stracke et al. (2007), has been shown to be deficient in flavonols, but to accumulate anthocyanins and PAs. This study also revealed, that structural closely related R2R3–MYBs (from the same subgroup), which regulate shared parts of the same flavonoid biosynthesis pathway, have redundant molecular functions, but different biological functions due to their divergent expression patterns. Seedlings mutated for *pfg1-3* still accumulate wild-type levels of anthocyanins (Stracke et al. 2007), suggesting normal expression levels of *EBGs* such as *CHS* that are required for anthocyanin biosynthesis in this triple mutant. Some studies have suggested regulation of *EBGs* such as *CHS* and *CHI* by a PAP1-containing MBW complex (Borevitz et al. 2000; Tohge et al. 2005), while other studies have suggested that PAP1- and TT2-containing MBW complex might not be essential for the transcription of the *EBGs* (Nesi et al. 2000, 2001; Gonzalez et al. 2008; Xu et al. 2013).

We aimed to determine whether the expression of these *EBGs* is dependent on other flavonoid R2R3–MYBs, which would imply that the postulated division of labour between the TTG1-dependent and TTG1-independent branches is not complete. To precisely assess the functions of different flavonoid R2R3–MYBs, we generated and analyzed multiple *R2R3–MYB* loss-of-function mutants that regulate multiple branches of the flavonoid biosynthesis pathway. We generated a *pfg1 pfg2 pfg3 tt2* quadruple mutant (termed *pfg1-3 tt2*) and a *pfg1 pfg2 pfg3 pap1 pap2 pap3 pap4* septuple mutant (termed *pfg1-3 pap1-4*) to examine flavonol, anthocyanin and PA production, to study the control of the *EBGs* by PFGs, PAPs and TT2.

## Materials and methods

### Plant material

The *A. thaliana* accessions Columbia-0 (Col-0) and Nössen-0 (Nö-0) are the genetic background of the *r2r3-myb* mutants and thus were used as wild-type controls. We used an existing *myb* sextuple mutant *myb11-12-111-75-90-114 (pfg1-3 pap1 pap2 pap4)* (Naik et al. 2021) as background for CRISPR/Cas9-mediated mutagenesis of *PAP3/MYB113*, resulting in a *pfg1-3 pap1-4* septuple mutant. A *myb11-12-111 (pfg1-3)* triple mutant (Stracke et al. 2007) was used as background for the CRISPR/Cas9 mediated knock-out of *TT2/MYB123*, resulting in a *pfg1-3 tt2* quadruple mutant.

### CRISPR/Cas9-mediated knock-out of *PAP3* and* TT2*

For the generation of a *pap3* knock-out, the binary expression vector pDE–Cas9, which contains *A. thaliana* codon-optimized Cas9 and Gateway destination sequences (Fauser et al. 2014), was used. For *TT2* mutagenesis, the vector XNG–Cas9 (Niu et al. 2020) was used. Specific sgRNAs were designed to target *PAP3* (5’-TATTTCCTAGAAGTTTATGA-3’) and *TT2* (5’-TAAGACCGGGGATAAAGCGC-3’). For oligo annealing, the forward and reverse oligos (40 µM each) were denatured in annealing buffer (125 mM NaCl, 25 mM Tris–HCl pH 6.8, 1 mM EDTA) at 95 °C for 5 min and then gradually cooled down to 5 °C. The annealed oligos were then inserted into the appropriate vectors. The T-DNAs from the resulting plasmids were transferred into *A. thaliana* using *Agrobacterium tumefaciens* GV3101 pMP90 (Koncz and Schell 1986) via the floral dip method (Clough and Bent 1998). Genomic DNA was extracted from selected transformants and suitable targeted mutants were identified by PCR and subsequent Sanger sequencing.

### Identification of wild-type and mutant alleles (genotyping)

Genomic DNA was extracted from the plants, and the alleles were identified by PCR using the genotyping primers listed in Table S1. Alleles that could only be distinguished by single nucleotide polymorphisms (SNPs) were identified by Sanger sequencing of the PCR products.

### Extraction and quantification of soluble and insoluble proanthocyanidins

Mature dry seeds were homogenized with 1 mm zirconium beads in an acidic methanol–acetone solution (40% methanol, 32% acetone, and 0.05% trifluoroacetic acid) at 30 Hz using a TissueLyser (Retsch). The samples were incubated on a shaker at 26 °C and 1200 rpm for 15 min. After sedimentation, the soluble PAs in the supernatant were saved, while the insoluble PAs in the sediment were resuspended in 200 µL of acidic methanol. Both fractions were then mixed with 1.2 mL acidic butanol (1.85% HCl in butanol) and 40 µL of ferric reagent (2% NH_4_FE(III)SO_4_, 7.4% HCl). Samples were then incubated at 95 °C for 20 min and sedimented. The soluble and insoluble PAs were quantified photometrically at a wavelength of 550 nm. PA content was calculated using a cyanidin standard.

### Detection and quantification of anthocyanins

Seedlings were grown from seeds on filter paper soaked with 3 ppm norflurazon (NFZ) and 4% sucrose under 16 h light per day. Visible anthocyanin accumulation was documented in 3-day-old seedlings by photography. For anthocyanin quantification, 3-day-old seedlings were treated with 1 mL of acidic methanol (1 N HCl in methanol) and incubated overnight. Samples were sedimented and the absorbance of the supernatant was measured at 530 nm and 657 nm. The anthocyanin content was calculated using the formula A530—0.25 × A657 * weight (mg)^−1^, as described in Mehrtens et al. (2005).

For detection and quantification of anthocyanins induced by high light stress, plants were grown on soil under short-day conditions (8 h light per day). After 32 days, the plants were transferred to high light conditions (16 h light per day with 1200 µmol photons m^−2^ s^−1^) for further 3 days. Extraction and quantification of the anthocyanins was performed as described above.

For detection of anthocyanins in stems, the plants were grown on soil under short-day conditions (8 h light per day). After 32 days, the plants were transferred to long-day conditions (16 h light per day) and observed after a further 20 days.

### High-performance thin-layer chromatography (HPTLC) of flavonol glycosides

Methanolic extracts were prepared from 3-day-old, light-grown seedlings that had been grown on 0.5 MS plates supplemented with 4% sucrose, rosette leaves and stems from plants grown as described above, and dry seeds. About 50–100 mg harvested plant material was weighed and homogenized in 80% methanol. After incubation at 70 °C for 15 min, the samples were centrifuged at 16,000 g for 10 min. The resulting supernatants were vacuum dried at 60 °C in a SpeedVac. The dried pellets were dissolved in 1 µL of 80% methanol per mg fresh weight. 3 µL of each sample (0.5 µL in case of dry seed samples and 1 µL in case of seedlings grown on 4% sucrose) were analyzed by HPTLC on a silica gel 60 plate (Merck) used as the stationary phase and a system of ethyl acetate, formic acid, acetic acid and water (100:26:12:12, by vol.) as the mobile phase in a closed glass tank. Separated phenylpropanoid compounds were stained by spraying a 1% diphenylboric acid 2-aminoethylester (DPBA) solution in methanol, and a 5% PEG4000 methanolic solution, as described by Sheahan and Rechnitz (1992). Visualization of flavonol derivatives was performed photographically under UV light (365 nm).

### Gene expression analysis using quantitative RT-PCR

To analyse R2R3–MYB-dependent expression of structural flavonoid biosynthesis genes, total RNA was extracted from tissues that accumulate flavonols and either PAs or anthocyanins. Siliques (including developing seeds) measuring 15 mm in length are known to express *EBGs* and PA-branch-specific *LBGs* (e.g., *CHS*, *TT2, ANR* and *TT10*), as well as exhibiting significant flavonol (Stracke et al. 2010b) and PA accumulation (Kleindt et al. 2010). Three-day-old seedlings grown on media containing 4% sucrose are known to accumulate flavonols (Appelhagen et al. 2014) and high levels of anthocyanins and show high expression of *EBGs* and anthocyanin biosynthesis-related *LBGs* (e.g., *CHS* and *DFR*) (Kubasek et al. 1992).

Three biological replicates were harvested for each genotype and RNA was extracted using a Spectrum™ Plant Total RNA Kit (Sigma-Aldrich). cDNA was synthesized using a ProtoScript® II First Strand cDNA Synthesis Kit [New England Biolabs (NEB)]. The qRT-PCR was performed in a 10 µL volume using the Luna Universal qPCR Master Mix (NEB) in the CFX96 Real-Time PCR Detection System (Bio-Rad). For each biological replicate, two technical replicates were performed using the qRT-PCR primers given in Table S1. Measurements were performed using the Bio-Rad Software CFX Maestro V.4.1.2433.1219. Normalization was done using the ΔΔCt method relative to the Col-0 wild type and the geometric mean of three reference genes (*MONESIN SENSITIVITY1 (MON1, At2g28390)*, *UBIQUITIN CONJUGATING ENZYME9* (*UBC9*, *At4g27960*) and *TAP42 INTERACTING PROTEIN OF 41KDA* (*TIP41*, *At4g34270*)). Error bars represent the standard error of the mean (SE) of log₂-transformed ΔΔCt values. Fold-change values were back-transformed to the linear scale for plotting, resulting in asymmetric error bars.

Representative *EBGs* and *LBGs* were selected to analyse R2R3–MYB-dependent flavonoid biosynthesis gene expression. The *EBGs* were *CHS* (which encodes the key enzyme in flavonoid biosynthesis), *CHI* (which encodes a representative enzyme involved in the formation of dihydroflavonols), and *FLS1* (which encodes a representative enzyme specific to the flavonol-branch). The *LBGs* were *ANS* (which encodes a representative enzyme required for anthocyanin and PA biosynthesis), and *ANR* (which encodes a representative enzyme required only for PA biosynthesis).

## Results

We generated a *pfg1-3 tt2* quadruple mutant and a *pfg1-3 pap1-4* septuple mutant, using crosses and targeted genome editing. In comparison with the *pfg1-3* mutant, we determined their genomic backgrounds and examined flavonoid accumulation patterns in different flavonoid-containing organs.

### Generation and genomic characterization of *myb* multiple mutants

**Fig. 1:**
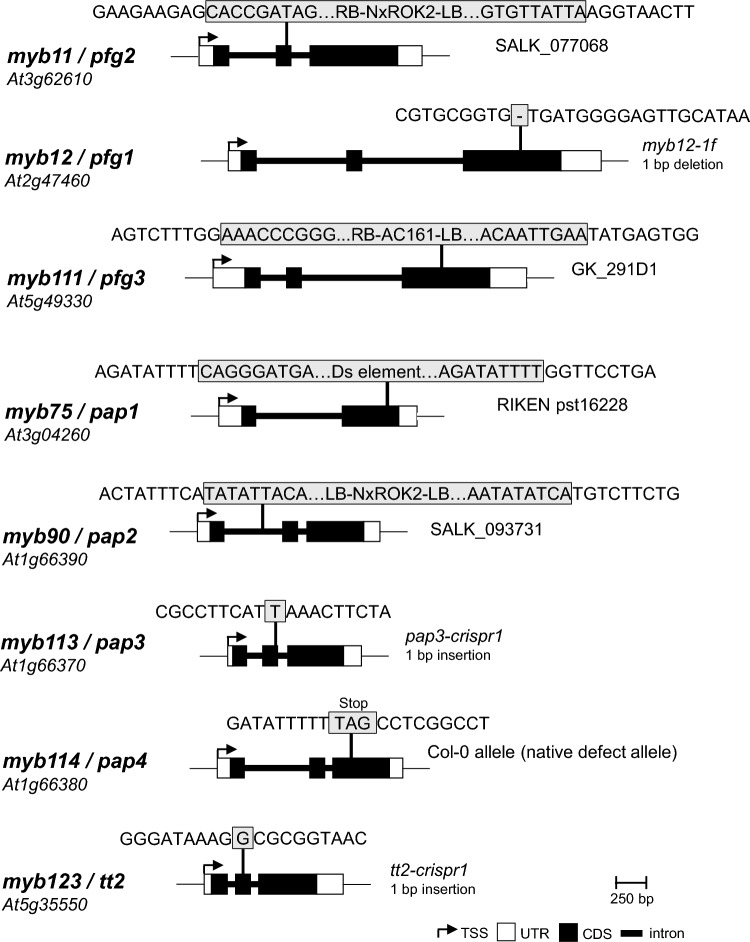
Genomic characterization of the *A. thaliana* flavonoid *r2r3*-*myb* mutant alleles used in this study. The schematic drawings display the respective mutations in the gene body. The exon–intron structures of the wild-type genes are indicated. The positions of the mutations (insertions of T-DNA or Ds element, 1 bp insertions/deletions or SNP resulting in a premature STOP codon) are indicated by a vertical line. The gene architectures are taken from the Col-0 genome, except for *pap4*, which is taken from the L*er*-0 genome. TSS, transcription start site; UTR, untranslated region; CDS, coding sequence

Because the genes *PAP2 (At1g66390), PAP3 (At1g66370)* and *PAP4 (At1g66380)* are consecutive genes on chromosome 1 (Fig. 1), it is almost impossible to find recombination events between them. Such recombination events would be required to generate a mutant of all three genes by crossing of existing mutants. We, therefore, used an existing, *pfg1 pfg2 pfg3 pap1 pap2 pap4* sextuple mutant (Naik et al. 2021) as background for CRISPR/Cas9-mediated mutagenesis of *PAP3*, resulting in a *pfg1 pfg2 pfg3 pap1 pap2 pap3 pap4 (pfg1-3 pap1-4)* septuple mutant. This mutant lacks all known flavonol and anthocyanin regulators from the R2R3–MYB TF family. An existing *pfg1 pfg2 pfg3 (pfg1-3)* triple mutant (Stracke et al. 2007) was used as background for the CRISPR/Cas9 mediated knock-out of *TT2*, resulting in a *pfg1 pfg2 pfg3 tt2* quadruple mutant *(pfg1-3 tt2)*. The resulting mutants were characterized using PCR and Sanger sequencing of the amplicons. This confirmed the previously described mutant alleles of *pfg1-3* from the triple mutant, as well as the newly generated mutant alleles of *pap3* (*pap3-crispr1*) and *tt2* (*tt2-crispr1*). Allele *pap3-crispr1* contains a single base pair insertion in the second exon causing a premature stop-codon after 78 amino acids (aa) of the protein (Fig. 1; Fig. S1), and allele *tt2-crispr1* contains a single base pair insertion in the second exon resulting in a premature stop-codon after 73 aa of the protein (Fig. 1; Fig. S1). All mutant alleles used in this study are listed in Table S2.

### Phenotypic characterization of the flavonoid multiple *r2r3-myb* mutants

The flavonoid composition of the different multiple *r2r3*-*myb* mutants as well as the corresponding Col-0 and Nö-0 wild-types were characterized by examining the accumulation of the three relevant flavonoid classes: PAs, anthocyanins and flavonols. Our focus was on non-reproductive organs, as the regulation of flavonoid biosynthesis differs in these organs.

### Proanthocyanidins

**Fig. 2:**
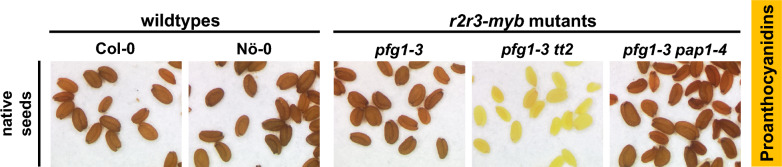
Proanthocyanidin accumulation in *A. thaliana* flavonoid multiple *r2r3-myb* mutants. Native seed color of wild-type and mutant seeds caused by PA accumulation (brown)

Accumulation of PAs in the testa result in the characteristic brown color of *A. thaliana* seeds, with higher PA levels resulting in a darker seed color. Native wild-type, *pfg1-3* and *pfg1-3 pap1-4* seeds show the characteristic brown seed color, while only *pfg1-3 tt2* seeds show a pale yellow color, indicating the lack of PAs (Fig. 2). Photometric quantification of the PA content supported this finding, as only the *pfg1-3 tt2* mutant shows a significantly reduced content of soluble and insoluble PAs (Fig. S2, raw data in Table S3). The residual calculated amount of PAs (partially) reflects a method-intrinsic background signal, which is also detected in the flavonoid-deficient *chs* mutant (Appelhagen et al. 2014), rather than actual PA accumulation (Fig. S3, raw data in Table S4). These findings indicate, that the *pfg1-3 tt2* regulator mutant is unable to form anthocyanins, whereas this ability is present in the other regulator mutants.

### Anthocyanins

**Fig. 3:**
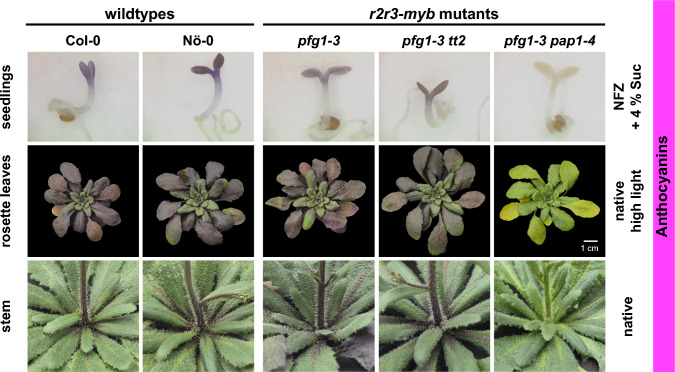
Anthocyanin accumulation in *A. thaliana* flavonoid multiple *r2r3-myb* mutants. Upper panel Norflurazon (NFZ)-bleached seedlings grown on 4% sucrose for induction of anthocyanin accumulation. Middle panel Native rosette of plants grown under high light for induction of anthocyanin accumulation. Lower panel Native stems of plants grown under high light for induction of anthocyanin accumulation

Sucrose-induced accumulation of anthocyanins leads to the red coloring of cotyledons and hypocotyls of *A. thaliana* seedlings, which can be seen in seedlings bleached with the herbicide norflurazon (NFZ). Both wild-types, *pfg1-3* and *pfg1-3 tt2* mutant seedlings accumulate anthocyanin (Fig. 3, top panel), while the *pfg1-3 pap1-4* seedlings do not show any visible red coloration, indicating the absence of anthocyanin pigments. Photometric quantification of anthocyanins (Fig. S4A) supports this observation with quantitative data and shows a significant induction of anthocyanin accumulation when grown on 4% sucrose in wild-types, the *pfg1-3* and the *pfg1-3 tt2* mutant (Fig. S4A, raw data in Table S5).

High-light induced anthocyanin accumulation was analyzed in rosette leaves (Fig. 3, middle panel) and stems of adult plants (Fig. 3, lower panel). As with seedlings, the *pfg1-3* and *pfg1-3 tt2* mutants exhibited wild-type-like anthocyanin accumulation, whereas no anthocyanins were observed in the *pfg1-3 pap1-4* mutant (Fig. 3, Fig. S4B, raw data in Table S6). These findings indicate that the *pfg1-3 pap1-4* mutant is unable to form anthocyanins, whereas the other mutants are capable of doing so.

### Flavonols

**Fig. 4:**
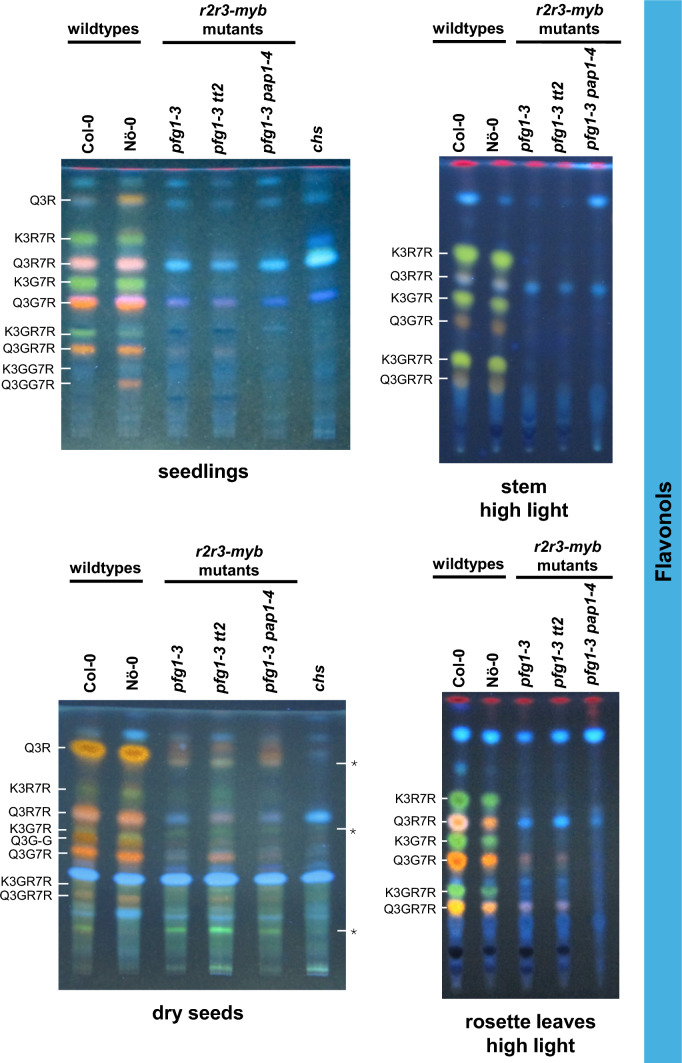
Flavonol accumulation in *A. thaliana* flavonoid multiple *r2r3-myb* mutants. Thin layer chromatography of methanolic extracts show the accumulation of flavonol glycoside derivatives in seedlings, in native stems and rosette leaves of plants grown under high light as well as in mature, dry seeds. Flavonol glycosides were identified from known literature (Stracke et al. 2007, 2010a, b). Quercetin (Q) derivatives appear orange and kaempferol (K) derivatives appear green under UV-light after staining with diphenylboric acid 2-aminoethylester (DPBA) and polyethylene glycole 4000 (PEG). Sinapic acid derivatives appear blue. G, glucose; R, rhamnose; *, “faint green” appearing non-flavonol flavonoids (see Fig. S6)

Flavonol accumulation analysis was done using high performance thin layer chromatography (HPTLC) of methanolic extracts. Hereby, kaempferol glycosides appear as green spots, while quercetin glycosides appear as orange/yellow spots. Blue spots are Brassicaceae-typical sinapate acid and sinapate ester derivatives. Flavonol glycoside accumulation was analyzed exemplarily in seedlings, rosette leaves, stems, and dry seeds (Fig. 4). In seedlings, the *pfg1-3* mutant shows the known absence of flavonol glycosides, which is also seen in the *pfg1-3 tt2* and *pfg1-3 pap1-4* mutant. The same observation was made for stems.

Analysis of flavonol accumulation in rosette leaves of plants grown under high light conditions, revealed a weak accumulation of the flavonol glycosides Q3G7R and Q3G7R in *pfg1-3* and *pfg1-3 tt2* mutants. This indicates PFG-independent flavonol accumulation. In contrast, no such accumulation was observed in the *pfg1-3 pap1-4* mutant, suggesting that PAPs are involved in the seen accumulation of flavonol derivatives.

## Discussion

The R2R3–MYBs that regulate flavonoid biosynthesis in *A. thaliana* can be classified into three branch-specific types (PFGs, PAPs, TT2) which regulate specific flavonoid branches forming flavonols, anthocyanins and PAs. Although the three branch-specific R2R3–MYB types have different sets of target genes, some of these genes may be regulated by more than one R2R3–MYB type, as known for the structural genes *DFR* and *ANS*, which are regulated by PAP-type R2R3–MYBs and TT2 (Borevitz et al. 2000; Nesi et al. 2000, 2001). We generated a set of multiple *R2R3–MYB* knock-out mutants to analyze metabolic differences, focusing on flavonol, anthocyanin and PA accumulation.

We examined the genomic regions of the existing and newly generated mutants using PCR and Sanger sequencing. While the known mutant alleles could be confirmed, sequence analysis revealed single base pair insertions in the newly generated knock-out alleles of *TT2 (tt2-crispr1)* and *PAP3 (pap3-crispr1)*. These insertions lead to premature stop-codons, resulting in truncated translational products of 73 aa (of 258 aa) for TT2 and 78 (of 246 aa) aa for PAP3. The two HTH motifs of the R2 and R3 repeat of PAP3 are predicted to be between the amino acids 33–57 and 85–108. A truncated protein of 78 aa would lack the HTH motif of repeat R3 and the C-terminus of the protein. The two HTH motifs of TT2 are located between the amino acids 39–63, and between the amino acids 91–114. A truncated protein of 73 aa lacks the HTH motif of R3 and the protein’s C-terminus. As the DNA binding in R2R3–MYB TFs relies on the third helices of each repeat (Ogata et al. 1994; Jia et al. 2004), the truncated TT2 and PAP3 proteins are predicted to (partially) lack DNA binding functionality as well the ability to activate their target genes, as the transactivation domain of R2R3–MYB proteins is located in the C-terminal part of the protein, behind the R2R3–MYB domain (Goff et al. 1991; Stracke et al. 2017; Morffy et al. 2024).

Previous studies have shown that the knock-out of all R2R3–MYBs, regulating a flavonoid biosynthesis branch, does not inhibit other flavonoid biosynthesis branches. For instance, the *tt2* mutant still accumulates flavonols and anthocyanins and has wild-type levels of transcripts from *EBGs* (Nesi et al. 2000, 2001). In addition, the *pfg1-3* mutant still accumulates anthocyanins and PAs (Fig. 2; Fig. 3), although it displayed significant reduced transcript levels in the EGBs *CHS, CHI, F3´H* and *FLS1* (Stracke et al. 2007). Nevertheless, there have also been hints towards the incompleteness of this model. For instance, studies published by Tohge et al. (2005) and Borevitz et al. (2000) showed a significant increase in the transcript levels of the *EBGs CHS* and *CHI* in leaves and roots of 3-week-old PAP1-overexpression plants, as well as vegetative leaves of 6-week-old plants, respectively. This is also supported by the work of Guo et al. (2023), in which the *EBGs CHS*, *CHI, F3H* and *F3’H* as well as the *LBGs DFR* and *ANS*, were identified as PAP1 target genes.

These findings regarding the overexpression of *PAP1* strengthen the hypothesis that not only the PFGs can activate the transcription of flavonoid *EBGs*, but also other TFs like PAP1 (in the context of an MBW complex). As the expression of *EBGs* is indispensable for anthocyanin and PA biosynthesis, these genes are likely regulated by TFs other than PFG1-3 which are non-functional in the *pfg1-3* mutant. As the *pfg1-3 tt2* mutant is the only one of the analyzed mutants that shows a significant decrease in the amount of both, soluble and insoluble PAs compared to the wild-type (Fig. S2), it is suggested that TT2 (as part of an MBW complex) is sufficient to activate the transcription of all the structural genes necessary for PA biosynthesis in seeds, including the *EBGs* needed for dihydroflavonol formation. This hypothesis is supported by gene expression analyses using qRT-PCR (Fig. S5A, raw data in Table S7), which show that the *EBGs CHS* and *CHI* are downregulated in siliques of the *pfg1-3 tt2* mutant, compared to the *pfg1-3* mutant. The absence of PAs which is observed in this study for the *pfg1-3 tt2* mutant is attributed to the newly generated *tt2-crispr1* knock-out allele. This phenotype is consistent with those reported for other *tt2* alleles (Nesi et al. 2001; Chen et al. 2012; Appelhagen et al. 2014), particularly the classical *tt2-1* allele (Koornneef 1990).

Although the amount of cyanidin is greatly reduced in the *pfg1-3 tt2* mutant compared to the wild-type, a slight increase compared to the completely flavonoid-deficient *chs* mutant was observed (Fig. S3) and might be explained by the presence of other flavonoids in the *pfg1-3 tt2* mutant, which are absent in the *chs* mutant, as also seen in HPTLC analyses of dry seeds (Fig. 4). Here, some "faint green" appearing metabolites were present in the analyzed multiple *r2r3-myb* mutants and the wild-type, but not in the *chs* mutant. The absence of these metabolites in seeds of the *chs* mutant as well as their visibility under UV-light without flavonol-staining (Fig. S6), suggests that they are flavonoids but not flavonol derivatives. However, the identity of these compounds remains to be clarified. The residual amounts of cyanidin in the *chs* mutant (Fig. S3) reflect the baseline of the conducted assay (Appelhagen et al. 2014).

Quantification of the anthocyanins in the *r2r3-myb* mutant lines revealed a similar result. While the *pfg1-3* and *pfg1-3 tt2* mutants accumulate anthocyanins at wild-type levels, only the *pfg1-3 pap1-4* mutant shows a significant decrease. This phenotype was evident in both analyzed samples, seedlings grown on high sucrose and rosette leaves of mature plants grown under high light conditions. The absence of anthocyanins in the *pfg1-3 pap1-4* mutant is attributed to the knock-out of all four *PAP genes*. This phenotype is consistent with the previously reported phenotype for an RNAi *PAP1-4* knock-down mutant (originally named MybRNAi), showing strong anthocyanin deficiencies (Gonzalez et al. 2008). These findings suggest that all genes necessary for anthocyanin biosynthesis can be activated by the PAP TFs, including the *EBGs* needed for dihydroflavonol formation. This hypothesis is supported by gene expression analyses using qRT-PCR (Fig. S5B, raw data in Table S8), which show that the *EBGs CHS* and *CHI* are downregulated in seedlings of the *pfg1-3 pap1-4* mutant, compared to the *pfg1-3* mutant.

**Fig. 5:**
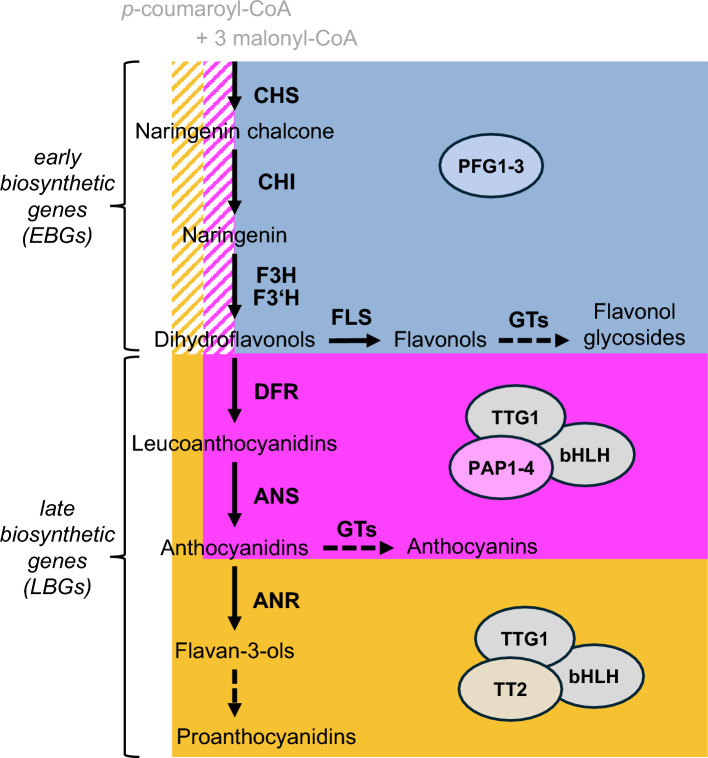
Adapted model of the regulation of flavonoid biosynthesis in *A. thaliana*. Black arrows indicate conversions by a single enzyme. Dashed arrows indicate multiple modification steps. Colored boxes in blue, purple and yellow highlight genes which have been confirmed to be regulated by PFGs, PAPs and TT2, respectively. Striped colored boxes represent regulatory interactions that are so far only supported by genetic and metabolic evidence and do not represent quantitative regulatory strength. PFG1-3, PRODUCTION OF FLAVONOL GLYCOSIDE1-3; PAP1-4, PRODUCTION OF ANTHOCYANIN PIGMENT1-4; TTG1, TRANSPARENT TESTA GLABRA1; bHLH, basic helix–loop–helix; TT2, TRANSPARENT TESTA2; CHS, chalcone synthase: CHI, chalcone isomerase; F3H, flavanone 3-hydroxylase; F3’H, flavonoid 3’-hydroxylase; FLS, flavonol synthase; DFR, dihydroflavonol 4-reductase; ANS, anthocyanidin synthase; ANR, anthocyanidin reductase; GT, glycosyl transferase

In summary, the detailed evaluation of published data from various authors and the study of a set of specific multiple *r2r3-myb* mutants allows to describe a more precise model of flavonoid biosynthesis regulation: *EBGs* are regulated not only by PFGs but also by PAP and TT2 TFs within their respective MBW complexes. *EBGs* of the flavonoid biosynthesis pathway, defined as genes that are expressed before visible pigmentation occurs (Lepiniec et al. 2006; Mao et al. 2025), include *CHS*, *CHI*, *F3H*, *F3’H* and *FLS1*. Expression of these genes *EBGs* (except *FLS1*) is needed for the formation of all three flavonoid classes (flavonols, anthocyanins, and PAs) and is regulated by all three R2R3–MYB types: PFGs, PAPs, and TT2. The structural *EBG FLS1* possess an exception as it is not shared between the three branches, being unique to the flavonol branch and is only regulated by the PFGs. In contrast to *EBGs*, *LBGs* are expressed after the initial reactions and are involved in visible pigment formation. These *LBGs* show a more specialized regulation: shared *LBGs* (*DFR*, *ANS*) between the anthocyanin and PA branch are regulated by both, PAPs and TT2, as supported by qRT-PCRs, which show that *ANS* is downregulated in the same way, in the *pfg1-3 tt2* and *pfg1-3 pap1-4* mutant (Fig. S5). The expression of genes encoding glycosyl transferases (GTs), which are described to be specific for the decoration of anthocyanidins (Zhu et al. 2025), is somehow similar to that of *FLS1*. They are only activated by PAPs. Potentially existing GTs which are not specific to one specific flavonoid class are not included in this pathway but might behave similar to genes shared between two or three branches. *LBGs* specific only for the PA branch [e.g., anthocyanidin reductase (ANR)] are regulated only by TT2 (Fig. 5).

Alongside the results described above, which are the basis for the proposed model, we also observed unexpected findings regarding the flavonol accumulation in the analyzed mutant lines, even though non-functional PFG TFs. While flavonol biosynthesis has been proposed to be regulated by the PFG TFs (Stracke et al. 2007), a lack of flavonol glycosides was expected in all analyzed mutant lines. However, we detected flavonol glycoside accumulation in dry seeds of all analyzed *r2r3-myb* mutant lines and in high light grown rosette leaves of the *pfg1-3* and *pfg1-3 tt2* mutants (Fig. 4). The presence of flavonols in the *pfg1-3* mutant does not align with previous findings (Stracke et al. 2007, 2010a), in which no flavonol glycosides were detected in this mutant. This discrepancy is presumably due to differences in the analyzed developmental stages and mainly the growth conditions. While Stracke et al. (2007, 2010a) analyzed 5-day-old seedlings and different plant organs, including siliques and rosette leaves of 8-week-old plants, grown under normal light and short- and long-day conditions, our present study used high-light conditions for the rosette leaves and dry seeds. While flavonol glycosides were detected within seeds of all analyzed mutants (*pfg1-3*, *pfg1-3 tt2*, and *pfg1-3 pap1-4*), mainly the derivatives Q3GR7R and Q3G7R accumulated in rosette leaves of the *pfg1-3* and *pfg1-3 tt2* mutants under high-light conditions, but were absent in the *pfg1-3 pap1-4* mutant. These findings indicate that the enzymatic activities leading to flavonol accumulation are present in the analyzed tissues (seeds, rosette leaves) and are, therefore, not exclusively regulated by the PFGs. One possible explanation is the regulation of *EBGs* by PAPs, which are highly expressed under high-light conditions and TT2, which is highly expressed in seeds. The enzymatic activities leading to the formation of flavonols include those of *CHS, CHI, F3H,* and *F3’H* and *FLS1*(Borevitz et al. 2000; Rowan et al. 2009). The first four genes (leading to the formation of dihydroflavonols) are proposed to be regulated by PAPs and TT2 in addition to the PFGs. The structural gene *FLS1*, however, is thought to be regulated only by the PFG TFs, as it is specific to the flavonol branch of flavonoid biosynthesis and not shared between the three branches. This hypothesis is supported by the study of Stracke et al. (2007) demonstrating a strong reduction of *FLS1* transcript levels in *pfg1-3* mutant seedlings. Dismissing the regulation of *FLS1* by PAPs and TT2, the *FLS1* is unlikely to be expressed in seeds and rosette leaves of the mutant lines with the *pfg1-3* background. However, the accumulation of flavonol glycosides in seeds and rosette leaves hints towards FLS activity. Assuming that *FLS1* is not expressed, the observed accumulation of flavonol glycosides could be explained by the side activities of 2-oxoglutarate-dependent dioxygenase (2ODDs) enzymes involved in the flavonoid biosynthesis. The 2-ODD-type enzymes F3H, FLS1 and ANS are closely related by sequence and all catalyze the oxidation of the C-ring. These enzymes have been shown to catalyze each other’s reactions, at least in part. In particular, *A. thaliana* F3H and ANS have been shown to exhibit FLS activity in vitro and *in planta*, enabling the formation of quercetin and kaempferol from the respective dihydroflavonols (Turnbull et al. 2004; Stracke et al. 2009; Schilbert et al. 2024). As both enzymes, F3H and ANS, are essential for anthocyanin and PA biosynthesis and regulation by PAP TFs and TT2 (in the absence of functional PFGs) is suggested, the formation of some levels of flavonol glycoside derivatives can be explained by the upregulation of *F3H* and *ANS* under high-light or sugar stress, or in seeds, resulting in high enzyme levels and thus higher (side) activity. The accumulation of flavonols in rosette leaves of the *pfg1-3* and *pfg1-3 tt2* mutant can, therefore, be explained by the high-light driven highly expressed PAP TFs, which activate not only the *EBGs* leading to the formation dihydroflavonols, but also the *LBGs F3H* and *ANS* which exhibit FLS activity.

The biosynthesis of Q3G7R which was detected in rosette leaves of the *pfg1-3* and *pfg1-3 tt2* mutants requires, beside the flavonol formation, two further glycosylation steps: 3-*O*-glucoslyation and 7-*O*-rhamnosylation of quercetin. These reactions are catalyzed by the UDP-glycosyltransferases (UGTs) UGT78D2 and UGT89C1, respectively (Saito et al. 2013). UGT78D2 has been shown to accept both flavonols and anthocyanins as substrates, making the regulation of the *UGT78D2* gene by PFG and PAP TFs highly plausible (Pourcel et al. 2010; Kim et al. 2013). Contrary to this, UGT89C1 accepts only 3-*O*-glycosylated flavonols as substrates and not anthocyanins (Yonekura-Sakakibara et al. 2007), rendering regulation by PAP TFs unlikely. Although UGT89C1, which is known to catalyse the 7-*O*-rhamnosylation of Q3G (Yonekura-Sakakibara et al. 2007), is unlikely regulated by the PAP TFs, the formation of Q3G7R based on PAP activity in the *pfg1-3 tt2* mutant is still possible. This is because several other UGTs have been reported to accept both, flavonols and anthocyanins, as substrates (Ford et al. 1998; Vogt and Jones 2000). Furthermore, an alternative biosynthetic route from Q3G to Q3G7R and Q3GR7R has already been proposed (Ishihara et al. 2016), but, to our knowledge, has not yet been identified. The presence of flavonols in rosette leaves of the *pfg1-3* and *pfg1-3 tt2* mutant grown under high-light conditions can, therefore, be explained by the PAP-mediated regulation of *EBGs* leading to the formation of dihydroflavonols, the side activities of F3H and ANS, and UGTs that accept both flavonols and anthocyanins as substrates. This hypothesis fits the finding that flavonol derivatives are completely absent in the *pfg1-3 pap1-4* mutant. Although TT2-mediated regulation of the *EBGs* (leading to the formation of dihydroflavonols) and *F3H* and *ANS* (which exhibit FLS side activity) is also suggested in this study, no accumulation of flavonol glycoside derivatives was found in the *pfg1-3 pap1-4* mutant in seedlings, rosette leaves and stems (Fig. 4). This finding can be explained by the spatio-temporal expression pattern of *TT2*, which is exclusively expressed in developing seeds, but not in vegetative parts, such as rosette leaves (Nesi et al. 2001). Consequently, TT2-mediated activation of *EBGs* is implausible in rosette leaves of the *pfg1-3 pap1-4* mutant, which explains the complete absence of flavonols in the rosette leaves of this mutant.

The detected accumulation of flavonol glycoside in dry seeds of all analyzed mutant lines can be partially explained accordingly. Flavonol accumulation in the *pfg1-3* and *pfg1-3 pap1-4* mutant could be explained by the activation of the *EBGs* necessary for dihydroflavonol biosynthesis by TT2, and the FLS side activities of the F3H and ANS enzymes. However, the *pfg1-3 tt2* mutant also accumulates some flavonol glycosides, which suggest that regulation by TT2 is not a contributing factor in this instance. As anthocyanin accumulation is absent in wild-type seeds (Routaboul et al. 2006), but PAP1 transcripts have been detected in developing seeds (Schmid et al. 2005; Kleindt et al. 2010; Bhargava et al. 2010), it is conceivable, that PAP1 could be the factor in developing seeds activating the *EBGs* in the absence of other R2R3–MYB *EBG* activators. Again, taken the 2ODDs side activities, this regulation would lead to the formation of flavonols. The absence of anthocyanins in seeds despite PAP1 activity in preceding developmental stages, may be due to differential spatial gene activity in the seed (Lepiniec et al. 2006) or to metabolic flux channeling towards flavonols, rather than anthocyanins in the seed (Jiang et al. 2020).

Overall, the findings concerning the accumulation of anthocyanins and proanthocyanidins in the analyzed mutant lines suggest, that both, TT2 and PAPs (as part of MBW complexes), have the potential to activate the transcription of the *EBGs*. This includes the *EBGs* that lead to the formation of dihydroflavonols. The basis for the different regulations of the *EBG FLS1* remains to be discovered.

Our findings confirm the results previously published by Borevitz et al. (2000), Tohge et al. (2005) and Guo et al. (2023), who proposed that the *EBGs* of flavonoid biosynthesis are potential target genes of PAP1. Our findings indicate that the traditional model of separate, branch-specific MYB regulators in flavonoid biosynthesis is oversimplified. We, therefore, hypothesize that both the PAP TFs and TT2 may be capable of activating early and late biosynthetic genes of flavonoid biosynthesis. To confirm this, whole transcriptome analyses should be performed with the presented *r2r3-myb* mutant set, to identify the specific TT2 and PAP regulons.

## Supplementary Information

Below is the link to the electronic supplementary material.

Supplementary file1 (DOCX 58 KB)

Supplementary file2 (DOCX 1061 KB)

## Data Availability

All data supporting the findings of this study are available within this paper and its supplementary information. The mutants generated in this work will be made available to the scientific community via the Nottingham Arabidopsis Stock Center (NASC, www.arabidopsis.info).

## Abbreviations

*EBG*s: Early biosynthesis genes
*LBG*s: Late biosynthesis genes
TF: Transcription factor
