# Supplementary material for: Combined R2R3–MYB transcription factor mutants reveal the regulatory structure of the Arabidopsis thaliana flavonoid biosynthesis pathway
Source: Planta. 2026 Feb 6;263(3):70. doi: 10.1007/s00425-026-04938-8 (PMC12881018; doi:10.1007/s00425-026-04938-8)
Supplement: Supplementary file 1 — Supplementary file1 (DOCX 58 KB) [file 425_2026_4938_MOESM1_ESM.docx]

**Supplementary Tables**

**Table S1** Primers used for genotyping and gene expression analyses.

| **Gene** | **Primer name** | **Sequence (5’ to 3’)** | **Primer usage description** |
| --- | --- | --- | --- |
| *TT2* | B050 | AATCTATTCTCAACACAACACT | *TT2* genotyping primer |
|  | RS1920 | GAGGTTTGAGTTCCAATGATTC | *TT2* genotyping primer |
| *PFG2* | RS537 | TACAGTACTCTGTCTCTCGACTATC | *PFG2* genotyping primer |
|  | RS538 | TCTCCCTCTCCATTCTCTATTGTC | *PFG2* genotyping primer |
|  | RS630 | GCGTGGACCGCTTGCTGCAACTCTCTCAGG | SALK T-DNA LB genotyping primer |
| *PFG1* | RS545 | GGAAGCCATCCATCTCTCAAGACG | *PFG1* genotyping primer |
|  | FM84 | CGACGCATCGTTAACTTGGTGGTC | *PFG1* genotyping primer |
| *PFG3* | RS316 | AATCTATGCCTTCACTGCCGTTTCC | *PFG3* genotyping primer |
|  | RS546 | CCAACAAGCTACTACAAAACCACA | *PFG3* genotyping primer |
|  | 8409 | ATATTGACCATCATACTCATTGC | GABI-KAT T-DNA LB genotyping primer |
| *PAP1* | B046 | GCTCTGATGAAGTCGATCTT | *PAP1* genotyping primer |
|  | B047 | ATCAAAACACACAAATTAAAAGG | *PAP1* genotyping primer |
|  | RS644 | TACCTCGGGTTCGAAATCGAT | RIKEN Ds-transposon genotyping primer |
| *PAP2* | B120 | GTTGACATTAATTATACCAACCAAGAC | *PAP2* genotyping primer |
|  | B121 | TGGTATGTTACACACACAGATATATTC | *PAP2* genotyping primer |
|  | RS630 | GCGTGGACCGCTTGCTGCAACTCTCTCAGG | SALK T-DNA LB genotyping primer |
| *PAP3* | B045 | ATGAGTGTTCCAGTAATTCTT | *PAP3* genotyping primer |
|  | RS298 | ATGGGCGAATCACCCAAAGGGTTG | *PAP3* genotyping primer |
| *PAP4* | RS1465 | TGGGCTAAATCGGTGCAGGA | *PAP4* genotyping primer |
|  | RS478 | GAGAAACCTCACAAACAAGAGAACAC | *PAP4* genotyping primer |
| *MON1* | RS2315 | TTGCTTACCACACGTTCAGATGCG | *MON1* qRT-PCR primer |
|  | RS2316 | AGAAGAACAGCCTCAAGGCGTACC | *MON1* qRT-PCR primer |
| *UBC9* | RS2311 | TTAACAGCA ATGGAAGCATCTGCC | *UBC9* qRT-PCR primer |
|  | RS2312 | TCAGGGACCAAAGGATCATCTGGG | *UBC9* qRT-PCR primer |
| *TIP4* | RS2309 | AAGCTCATGGTTCCTCCTCTTGCG | *TIP41* qRT-PCR primer |
|  | RS2310 | CAGTTGGTGCCTCATCTTCGCC | *TIP41* qRT-PCR primer |
| *CHS* | RS793 | TCAGGCGGAGTATCCTGACTA | *CHS* qRT-PCR primer |
|  | RS794 | CGTTTCCGAATTGTCGACTT | *CHS* qRT-PCR primer |
| *CHI* | RS1152 | CTCTCTTACGGTTGCGTTTTCG | *CHI* qRT-PCR primer |
|  | RS1153 | CACCGTTCTTCCCGATGATAGA | *CHI* qRT-PCR primer |
| *FLS1* | RS801 | CACATCGGCGATCAGATTC | *FLS1* qRT-PCR primer |
|  | RS802 | GGGAGGCTCCAAGAAAACC | *FLS1* qRT-PCR primer |
| *ANS* | RS1146 | GGCTGTGTTTTGTGAGCCACCA | *ANS* qRT-PCR primer |
|  | RS1147 | CCTTGGAGGAAACTTAGCCGGAGA | *ANS* qRT-PCR primer |
| *ANR* | G121 | AAGAAAACTGGACTGACGTTGAA | *ANR* qRT-PCR primer |
|  | G122 | AACACCTTCGAGATTGGGTAAC | *ANR* qRT-PCR primer |

**Table S2** *r2r3-myb* mutant alleles used in this study.

| **Mutant** | **Gene** | **Synonym** | **Allele** | **Mutation** | **Allele described in** | **Allele background** |
| --- | --- | --- | --- | --- | --- | --- |
| *pfg1-3* | *MYB11* | *PFG2* | SALK077068 | T-DNA Insertion | Stracke et al. 2007 | Col-0 |
|  | *MYB12* | *PFG1* | myb12-1f | Transposon footprint | Mehrtens et al. 2005 | Col-0 |
|  | *MYB111* | *PFG3* | GK291D1 | T-DNA Insertion | Stracke et al. 2007 | Col-0 |
| *pfg1-3 pap1-4* | *MYB11* | *PFG2* | SALK077068 | T-DNA Insertion | Stracke et al. 2007 | Col-0 |
|  | *MYB12* | *PFG1* | myb12-1f | Transposon footprint | Mehrtens et al. 2005 | Col-0 |
|  | *MYB111* | *PFG3* | GK291D1 | T-DNA Insertion | Stracke et al. 2007 | Col-0 |
|  | *MYB75* | *PAP1* | RIKEN pst16228 | Transposon | Teng et al. 2005 | Nö-0 |
|  | *MYB90* | *PAP2* | SALK093731 | T-DNA Insertion | Appelhagen et al. 2011 | Col-0 |
|  | *MYB113* | *PAP3* | *pap3-crispr1* | CRISPR/Cas9, 1 bp insertion | this study | Col-0 |
|  | *MYB114* | *PAP4* | *myb114 (Col-0)* | Pseudogene in Col-0 | Gonzalez et al. 2008 | Col-0 |
| *pfg1-3 tt2* | *MYB11* | *PFG2* | SALK077068 | T-DNA Insertion | Stracke et al. 2007 | Col-0 |
|  | *MYB12* | *PFG1* | myb12-1f | Transposon footprint | Mehrtens et al. 2005 | Col-0 |
|  | *MYB111* | *PFG3* | GK291D1 | T-DNA Insertion | Stracke et al. 2007 | Col-0 |
|  | *MYB123* | *TT2* | *tt2-crispr1* | CRISPR/Cas9, 1 bp insertion | this study | Col-0 |

**Table S3** Raw data of proanthocyanidin quantification in *r2r3-myb* mutants.

| **Genotype** | **Weight [mg]** | **OD550 - insoluble cyandin** | **OD550 - soluble cyanidin** | **insoluble cyanidin [µg/mg]** | **soluble cyanidin [µg/mg]** |
| --- | --- | --- | --- | --- | --- |
| Col-0 | 13.1 | 0.166 | 0.246 | 3.375 | 4.805 |
| Col-0 | 9.2 | 0.216 | 0.196 | 6.078 | 5.569 |
| Col-0 | 13.6 | 0.211 | 0.230 | 4.026 | 4.353 |
| Col-0 | 11.7 | 0.201 | 0.228 | 4.479 | 5.020 |
| Col-0 | 9.7 | 0.177 | 0.181 | 4.823 | 4.920 |
| Col-0 | 8.7 | 0.148 | 0.188 | 4.597 | 5.674 |
| Nö-0 | 11.8 | 0.178 | 0.247 | 3.985 | 5.354 |
| Nö-0 | 8.8 | 0.274 | 0.226 | 7.898 | 6.621 |
| Nö-0 | 13.0 | 0.370 | 0.267 | 7.076 | 5.220 |
| Nö-0 | 9.8 | 0.306 | 0.239 | 7.857 | 6.256 |
| Nö-0 | 12.1 | 0.313 | 0.253 | 6.499 | 5.338 |
| Nö-0 | 9.2 | 0.203 | 0.218 | 5.747 | 6.129 |
| *pfg1-3* | 10.9 | 0.172 | 0.142 | 4.185 | 3.540 |
| *pfg1-3* | 10.3 | 0.237 | 0.136 | 5.906 | 3.610 |
| *pfg1-3* | 11.7 | 0.280 | 0.143 | 6.060 | 3.318 |
| *pfg1-3* | 10.3 | 0.234 | 0.129 | 5.838 | 3.451 |
| *pfg1-3* | 12.1 | 0.264 | 0.145 | 5.550 | 3.247 |
| *pfg1-3* | 9.8 | 0.178 | 0.125 | 4.798 | 3.531 |
| *pfg1-3 tt2* | 9.7 | 0.009 | 0.019 | 0.767 | 1.008 |
| *pfg1-3 tt2* | 10.4 | 0.010 | 0.021 | 0.738 | 0.986 |
| *pfg1-3 tt2* | 8.2 | 0.009 | 0.016 | 0.907 | 1.107 |
| *pfg1-3 tt2* | 10.8 | 0.009 | 0.023 | 0.689 | 0.992 |
| *pfg1-3 tt2* | 10.8 | 0.010 | 0.023 | 0.710 | 0.992 |
| *pfg1-3 tt2* | 9.0 | 0.009 | 0.017 | 0.827 | 1.035 |
| *pfg1-3 pap1-4* | 8.3 | 0.134 | 0.250 | 4.423 | 7.697 |
| *pfg1-3 pap1-4* | 8.1 | 0.204 | 0.255 | 6.557 | 8.031 |
| *pfg1-3 pap1-4* | 7.2 | 0.206 | 0.211 | 7.441 | 7.604 |
| *pfg1-3 pap1-4* | 8.2 | 0.194 | 0.228 | 6.191 | 7.162 |
| *pfg1-3 pap1-4* | 7.7 | 0.179 | 0.217 | 6.137 | 7.293 |
| *pfg1-3 pap1-4* | 5.7 | 0.124 | 0.201 | 6.030 | 9.194 |

**Table S4** Raw data of proanthocyanidin quantification.

| **Genotype** | **Weight [mg]** | **OD550 - insoluble cyandin** | **OD550 - soluble cyanidin** | **insoluble cyanidin [µg/mg]** | **soluble cyanidin [µg/mg]** |
| --- | --- | --- | --- | --- | --- |
| Col-0 | 16.5 | 0.124 | 0.099 | 1.769 | 1.430 |
| Col-0 | 18.7 | 0.149 | 0.112 | 1.860 | 1.417 |
| Col-0 | 15.5 | 0.149 | 0.106 | 2.244 | 1.623 |
| Col-0 | 13.9 | 0.149 | 0.105 | 2.503 | 1.794 |
| Col-0 | 14.0 | 0.149 | 0.099 | 2.485 | 1.686 |
| Col-0 | 16.1 | 0.163 | 0.097 | 2.355 | 1.438 |
| *pfg1-3 tt2* | 10.3 | 0.016 | 0.040 | 0.488 | 1.009 |
| *pfg1-3 tt2* | 10.0 | 0.013 | 0.034 | 0.435 | 0.905 |
| *pfg1-3 tt2* | 13.1 | 0.019 | 0.053 | 0.435 | 1.016 |
| *pfg1-3 tt2* | 13.9 | 0.026 | 0.052 | 0.522 | 0.941 |
| *pfg1-3 tt2* | 13.9 | 0.022 | 0.058 | 0.458 | 1.038 |
| *pfg1-3 tt2* | 21.5 | 0.037 | 0.075 | 0.452 | 0.848 |
| *chs* | 16.2 | 0.014 | 0.032 | 0.282 | 0.531 |
| *chs* | 15.8 | 0.015 | 0.034 | 0.304 | 0.573 |
| *chs* | 17.8 | 0.017 | 0.041 | 0.295 | 0.596 |
| *chs* | 15.7 | 0.016 | 0.032 | 0.320 | 0.548 |
| *chs* | 14.6 | 0.016 | 0.033 | 0.344 | 0.605 |
| *chs* | 13.6 | 0.015 | 0.031 | 0.353 | 0.616 |

**Table S5** Raw data of relative anthocyanin content in seedlings of *r2r3-myb* mutants.

| **Genotype** | **Sucrose [%]** | **Weight [mg]** | **OD530** | **OD657** | **Dilution factor** | **Anthocyanin content** | **relative anthocyanin content / mg fresh weight** |
| --- | --- | --- | --- | --- | --- | --- | --- |
| Col-0 | 0.5 | 30 | 0.096 | 0.177 | 5 | 0.259 | 0.009 |
| Col-0 | 0.5 | 29 | 0.087 | 0.142 | 5 | 0.258 | 0.009 |
| Col-0 | 0.5 | 43 | 0.148 | 0.274 | 5 | 0.398 | 0.009 |
| Col-0 | 0.5 | 32 | 0.104 | 0.202 | 5 | 0.268 | 0.008 |
| Col-0 | 0.5 | 38 | 0.099 | 0.189 | 5 | 0.259 | 0.007 |
| Col-0 | 0.5 | 39 | 0.086 | 0.165 | 5 | 0.224 | 0.006 |
| Nö-0 | 0.5 | 22 | 0.081 | 0.135 | 5 | 0.236 | 0.011 |
| Nö-0 | 0.5 | 43 | 0.162 | 0.279 | 5 | 0.461 | 0.011 |
| Nö-0 | 0.5 | 44 | 0.113 | 0.212 | 5 | 0.300 | 0.007 |
| Nö-0 | 0.5 | 34 | 0.104 | 0.171 | 5 | 0.306 | 0.009 |
| Nö-0 | 0.5 | 31 | 0.105 | 0.178 | 5 | 0.303 | 0.010 |
| Nö-0 | 0.5 | 23 | 0.080 | 0.176 | 5 | 0.180 | 0.008 |
| *pfg1-3* | 0.5 | 64 | 0.165 | 0.277 | 5 | 0.479 | 0.007 |
| *pfg1-3* | 0.5 | 55 | 0.179 | 0.301 | 5 | 0.519 | 0.009 |
| *pfg1-3* | 0.5 | 59 | 0.190 | 0.318 | 5 | 0.553 | 0.009 |
| *pfg1-3* | 0.5 | 63 | 0.170 | 0.288 | 5 | 0.490 | 0.008 |
| *pfg1-3* | 0.5 | 56 | 0.133 | 0.231 | 5 | 0.376 | 0.007 |
| *pfg1-3* | 0.5 | 55 | 0.246 | 0.315 | 5 | 0.836 | 0.015 |
| *pfg1-3 tt2* | 0.5 | 55 | 0.252 | 0.331 | 5 | 0.846 | 0.015 |
| *pfg1-3 tt2* | 0.5 | 34 | 0.227 | 0.295 | 5 | 0.766 | 0.023 |
| *pfg1-3 tt2* | 0.5 | 55 | 0.202 | 0.273 | 5 | 0.669 | 0.012 |
| *pfg1-3 tt2* | 0.5 | 46 | 0.164 | 0.295 | 5 | 0.451 | 0.010 |
| *pfg1-3 tt2* | 0.5 | 52 | 0.174 | 0.278 | 5 | 0.523 | 0.010 |
| *pfg1-3 tt2* | 0.5 | 41 | 0.296 | 0.391 | 5 | 0.991 | 0.024 |
| *pfg1-3 pap1-4* | 0.5 | 24 | 0.032 | 0.115 | 5 | 0.016 | 0.001 |
| *pfg1-3 pap1-4* | 0.5 | 39 | 0.053 | 0.188 | 5 | 0.030 | 0.001 |
| *pfg1-3 pap1-4* | 0.5 | 29 | 0.041 | 0.136 | 5 | 0.035 | 0.001 |
| *pfg1-3 pap1-4* | 0.5 | 35 | 0.053 | 0.186 | 5 | 0.033 | 0.001 |
| *pfg1-3 pap1-4* | 0.5 | 32 | 0.032 | 0.109 | 5 | 0.024 | 0.001 |
| *pfg1-3 pap1-4* | 0.5 | 23 | 0.029 | 0.100 | 5 | 0.020 | 0.001 |
|  |  |  |  |  |  |  |  |
| Col-0 | 4 | 19 | 0.282 | 0.099 | 5 | 1.286 | 0.068 |
| Col-0 | 4 | 19 | 0.254 | 0.093 | 5 | 1.154 | 0.061 |
| Col-0 | 4 | 21 | 0.285 | 0.130 | 5 | 1.263 | 0.060 |
| Col-0 | 4 | 21 | 0.330 | 0.142 | 5 | 1.473 | 0.070 |
| Col-0 | 4 | 14 | 0.211 | 0.080 | 5 | 0.955 | 0.068 |
| Col-0 | 4 | 20 | 0.328 | 0.111 | 5 | 1.501 | 0.075 |
| Nö-0 | 4 | 20 | 0.250 | 0.095 | 5 | 1.131 | 0.057 |
| Nö-0 | 4 | 11 | 0.111 | 0.043 | 5 | 0.501 | 0.046 |
| Nö-0 | 4 | 18 | 0.266 | 0.085 | 5 | 1.224 | 0.068 |
| Nö-0 | 4 | 21 | 0.257 | 0.106 | 5 | 1.153 | 0.055 |
| Nö-0 | 4 | 11 | 0.152 | 0.052 | 5 | 0.695 | 0.063 |
| Nö-0 | 4 | 15 | 0.207 | 0.077 | 5 | 0.939 | 0.063 |
| *pfg1-3* | 4 | 23 | 0.359 | 0.128 | 5 | 1.635 | 0.071 |
| *pfg1-3* | 4 | 21 | 0.343 | 0.129 | 5 | 1.554 | 0.074 |
| *pfg1-3* | 4 | 31 | 0.539 | 0.204 | 5 | 2.440 | 0.079 |
| *pfg1-3* | 4 | 27 | 0.476 | 0.180 | 5 | 2.155 | 0.080 |
| *pfg1-3* | 4 | 22 | 0.368 | 0.126 | 5 | 1.683 | 0.076 |
| *pfg1-3* | 4 | 27 | 0.405 | 0.145 | 5 | 1.844 | 0.068 |
| *pfg1-3 tt2* | 4 | 24 | 0.442 | 0.091 | 5 | 2.096 | 0.087 |
| *pfg1-3 tt2* | 4 | 21 | 0.366 | 0.075 | 5 | 1.736 | 0.083 |
| *pfg1-3 tt2* | 4 | 28 | 0.320 | 0.066 | 8 | 2.428 | 0.087 |
| *pfg1-3 tt2* | 4 | 42 | 0.441 | 0.093 | 8 | 3.342 | 0.080 |
| *pfg1-3 tt2* | 4 | 38 | 0.412 | 0.084 | 8 | 3.128 | 0.082 |
| *pfg1-3 tt2* | 4 | 47 | 0.424 | 0.091 | 10 | 4.013 | 0.085 |
| *pfg1-3 pap1-4* | 4 | 17 | 0.026 | 0.094 | 5 | 0.013 | 0.001 |
| *pfg1-3 pap1-4* | 4 | 25 | 0.041 | 0.137 | 5 | 0.034 | 0.001 |
| *pfg1-3 pap1-4* | 4 | 25 | 0.040 | 0.150 | 5 | 0.013 | 0.001 |
| *pfg1-3 pap1-4* | 4 | 15 | 0.023 | 0.075 | 5 | 0.021 | 0.001 |
| *pfg1-3 pap1-4* | 4 | 25 | 0.036 | 0.114 | 5 | 0.038 | 0.002 |
| *pfg1-3 pap1-4* | 4 | 25 | 0.040 | 0.133 | 5 | 0.034 | 0.001 |

**Table S6** Raw data of relative anthocyanin content in rosette leaves of *r2r3-myb* mutants.

| **Genotype** | **Weight [mg]** | **OD530** | **OD657** | **Dilution factor** | **Anthocyanin content** | **relative anthocyanin content / mg fresh weight** |
| --- | --- | --- | --- | --- | --- | --- |
| Col-0 | 61 | 0.53 | 0.22 | 10 | 4.768 | 0.078 |
| Col-0 | 64 | 0.50 | 0.19 | 10 | 4.495 | 0.070 |
| Col-0 | 57 | 0.47 | 0.23 | 10 | 4.078 | 0.072 |
| Col-0 | 55 | 0.43 | 0.15 | 10 | 3.960 | 0.072 |
| Col-0 | 54 | 0.41 | 0.12 | 10 | 3.835 | 0.071 |
| Col-0 | 63 | 0.45 | 0.21 | 10 | 3.985 | 0.063 |
| Nö-0 | 98 | 0.83 | 0.34 | 10 | 7.458 | 0.076 |
| Nö-0 | 88 | 0.76 | 0.32 | 10 | 6.768 | 0.077 |
| Nö-0 | 85 | 0.62 | 0.26 | 10 | 5.583 | 0.066 |
| Nö-0 | 94 | 0.84 | 0.32 | 10 | 7.590 | 0.081 |
| Nö-0 | 81 | 0.84 | 0.34 | 10 | 7.590 | 0.094 |
| Nö-0 | 59 | 0.58 | 0.28 | 10 | 5.103 | 0.086 |
| *pfg1-3* | 83 | 0.70 | 0.28 | 10 | 6.263 | 0.075 |
| *pfg1-3* | 91 | 0.66 | 0.26 | 10 | 5.913 | 0.065 |
| *pfg1-3* | 66 | 0.48 | 0.14 | 10 | 4.408 | 0.067 |
| *pfg1-3* | 74 | 0.61 | 0.22 | 10 | 5.523 | 0.075 |
| *pfg1-3* | 90 | 0.68 | 0.29 | 10 | 6.030 | 0.067 |
| *pfg1-3* | 57 | 0.52 | 0.29 | 10 | 4.495 | 0.079 |
| *pfg1-3 tt2* | 85 | 0.60 | 0.31 | 10 | 5.175 | 0.061 |
| *pfg1-3 tt2* | 83 | 0.61 | 0.28 | 10 | 5.425 | 0.065 |
| *pfg1-3 tt2* | 93 | 0.67 | 0.35 | 10 | 5.803 | 0.062 |
| *pfg1-3 tt2* | 58 | 0.35 | 0.10 | 10 | 3.243 | 0.056 |
| *pfg1-3 tt2* | 75 | 0.47 | 0.23 | 10 | 4.163 | 0.056 |
| *pfg1-3 tt2* | 68 | 0.50 | 0.28 | 10 | 4.270 | 0.063 |
| *pfg1-3 pap1-4* | 51 | 0.07 | 0.25 | 10 | 0.033 | 0.001 |
| *pfg1-3 pap1-4* | 91 | 0.08 | 0.28 | 10 | 0.093 | 0.001 |
| *pfg1-3 pap1-4* | 68 | 0.07 | 0.27 | 10 | 0.027 | 0.000 |
| *pfg1-3 pap1-4* | 78 | 0.06 | 0.20 | 10 | 0.108 | 0.001 |
| *pfg1-3 pap1-4* | 66 | 0.07 | 0.27 | 10 | 0.035 | 0.001 |
| *pfg1-3 pap1-4* | 79 | 0.07 | 0.26 | 10 | 0.067 | 0.001 |

**Table S7** Raw mean Cq values of qRT-PCR from silique RNA.

| **Target gene** | **Genotype** | **Mean Cq** |
| --- | --- | --- |
| *ANR* | Col-0 | 23.95 |
| *ANR* | Col-0 | 24.53 |
| *ANR* | Col-0 | 24.60 |
| *ANR* | *pfg1-3* | 24.36 |
| *ANR* | *pfg1-3* | 24.41 |
| *ANR* | *pfg1-3* | 24.36 |
| *ANR* | *pfg1-3 tt2* | 33.13 |
| *ANR* | *pfg1-3 tt2* | 36.88 |
| *ANR* | *pfg1-3 tt2* | 34.44 |
| *CHI* | Col-0 | 24.13 |
| *CHI* | Col-0 | 24.42 |
| *CHI* | Col-0 | 24.74 |
| *CHI* | *pfg1-3* | 25.31 |
| *CHI* | *pfg1-3* | 25.28 |
| *CHI* | *pfg1-3* | 25.27 |
| *CHI* | *pfg1-3 tt2* | 28.83 |
| *CHI* | *pfg1-3 tt2* | 28.97 |
| *CHI* | *pfg1-3 tt2* | 28.77 |
| *CHS* | Col-0 | 23.22 |
| *CHS* | Col-0 | 23.53 |
| *CHS* | Col-0 | 23.92 |
| *CHS* | *pfg1-3* | 23.95 |
| *CHS* | *pfg1-3* | 24.02 |
| *CHS* | *pfg1-3* | 24.14 |
| *CHS* | *pfg1-3 tt2* | 29.65 |
| *CHS* | *pfg1-3 tt2* | 30.11 |
| *CHS* | *pfg1-3 tt2* | 29.48 |
| *FLS1* | Col-0 | 26.24 |
| *FLS1* | Col-0 | 26.54 |
| *FLS1* | Col-0 | 26.83 |
| *FLS1* | *pfg1-3* | 30.33 |
| *FLS1* | *pfg1-3* | 30.70 |
| *FLS1* | *pfg1-3* | 30.65 |
| *FLS1* | *pfg1-3 tt2* | 30.70 |
| *FLS1* | *pfg1-3 tt2* | 30.91 |
| *FLS1* | *pfg1-3 tt2* | 30.33 |
| *ANS* | Col-0 | 23.14 |
| *ANS* | Col-0 | 23.47 |
| *ANS* | Col-0 | 24.09 |
| *ANS* | *pfg1-3* | 23.77 |
| *ANS* | *pfg1-3* | 23.77 |
| *ANS* | *pfg1-3* | 23.73 |
| *ANS* | *pfg1-3 tt2* | 30.96 |
| *ANS* | *pfg1-3 tt2* | 32.44 |
| *ANS* | *pfg1-3 tt2* | 32.14 |
| *MON1* | Col-0 | 28.87 |
| *MON1* | Col-0 | 29.24 |
| *MON1* | Col-0 | 29.40 |
| *MON1* | *pfg1-3* | 29.15 |
| *MON1* | *pfg1-3* | 29.04 |
| *MON1* | *pfg1-3* | 29.07 |
| *MON1* | *pfg1-3 tt2* | 29.20 |
| *MON1* | *pfg1-3 tt2* | 29.46 |
| *MON1* | *pfg1-3 tt2* | 29.22 |
| *TIP41* | Col-0 | 26.31 |
| *TIP41* | Col-0 | 26.58 |
| *TIP41* | Col-0 | 26.92 |
| *TIP41* | *pfg1-3* | 26.48 |
| *TIP41* | *pfg1-3* | 26.47 |
| *TIP41* | *pfg1-3* | 26.47 |
| *TIP41* | *pfg1-3 tt2* | 26.61 |
| *TIP41* | *pfg1-3 tt2* | 26.70 |
| *TIP41* | *pfg1-3 tt2* | 26.47 |
| *UBC9* | Col-0 | 22.48 |
| *UBC9* | Col-0 | 22.90 |
| *UBC9* | Col-0 | 23.05 |
| *UBC9* | *pfg1-3* | 22.78 |
| *UBC9* | *pfg1-3* | 22.82 |
| *UBC9* | *pfg1-3* | 22.78 |
| *UBC9* | *pfg1-3 tt2* | 22.76 |
| *UBC9* | *pfg1-3 tt2* | 23.01 |
| *UBC9* | *pfg1-3 tt2* | 22.76 |

**Table S8** Raw mean Cq values of qRT-PCR from seedling RNA.

| **Target gene** | **Genotype** | **Mean Cq** |
| --- | --- | --- |
| *ANS* | Col-0 | 25.66 |
| *ANS* | Col-0 | 25.03 |
| *ANS* | Col-0 | 25.00 |
| *ANS* | Nö-0 | 24.12 |
| *ANS* | Nö-0 | 23.61 |
| *ANS* | Nö-0 | 23.13 |
| *ANS* | *pfg1-3* | 22.73 |
| *ANS* | *pfg1-3* | 23.05 |
| *ANS* | *pfg1-3* | 23.05 |
| *ANS* | *pfg1-3 pap1-4* | 33.88 |
| *ANS* | *pfg1-3 pap1-4* | 33.57 |
| *ANS* | *pfg1-3 pap1-4* | 33.58 |
| *CHI* | Col-0 | 26.16 |
| *CHI* | Col-0 | 25.33 |
| *CHI* | Col-0 | 25.85 |
| *CHI* | Nö-0 | 24.83 |
| *CHI* | Nö-0 | 24.59 |
| *CHI* | Nö-0 | 24.04 |
| *CHI* | *pfg1-3* | 24.51 |
| *CHI* | *pfg1-3* | 24.56 |
| *CHI* | *pfg1-3* | 24.37 |
| *CHI* | *pfg1-3 pap1-4* | 26.86 |
| *CHI* | *pfg1-3 pap1-4* | 26.87 |
| *CHI* | *pfg1-3 pap1-4* | 26.70 |
| *CHS* | Col-0 | 26.91 |
| *CHS* | Col-0 | 26.39 |
| *CHS* | Col-0 | 26.07 |
| *CHS* | Nö-0 | 24.89 |
| *CHS* | Nö-0 | 24.27 |
| *CHS* | Nö-0 | 23.82 |
| *CHS* | *pfg1-3* | 25.34 |
| *CHS* | *pfg1-3* | 25.49 |
| *CHS* | *pfg1-3* | 25.52 |
| *CHS* | *pfg1-3 pap1-4* | 31.27 |
| *CHS* | *pfg1-3 pap1-4* | 31.29 |
| *CHS* | *pfg1-3 pap1-4* | 31.30 |
| *FLS1* | Col-0 | 27.32 |
| *FLS1* | Col-0 | 26.81 |
| *FLS1* | Col-0 | 26.95 |
| *FLS1* | Nö-0 | 25.84 |
| *FLS1* | Nö-0 | 25.54 |
| *FLS1* | Nö-0 | 24.84 |
| *FLS1* | *pfg1-3* | 30.66 |
| *FLS1* | *pfg1-3* | 31.12 |
| *FLS1* | *pfg1-3* | 30.82 |
| *FLS1* | *pfg1-3 pap1-4* | 30.85 |
| *FLS1* | *pfg1-3 pap1-4* | 31.07 |
| *FLS1* | *pfg1-3 pap1-4* | 30.92 |
| *MON1* | Col-0 | 30.80 |
| *MON1* | Col-0 | 30.53 |
| *MON1* | Col-0 | 30.46 |
| *MON1* | Nö-0 | 29.87 |
| *MON1* | Nö-0 | 29.48 |
| *MON1* | Nö-0 | 29.41 |
| *MON1* | pfg1-3 | 29.37 |
| *MON1* | *pfg1-3* | 29.32 |
| *MON1* | *pfg1-3* | 28.80 |
| *MON1* | *pfg1-3 pap1-4* | 29.43 |
| *MON1* | *pfg1-3 pap1-4* | 29.09 |
| *MON1* | *pfg1-3 pap1-4* | 28.80 |
| *TIP41* | Col-0 | 28.12 |
| *TIP41* | Col-0 | 27.63 |
| *TIP41* | Col-0 | 27.61 |
| *TIP41* | Nö-0 | 27.23 |
| *TIP41* | Nö-0 | 26.92 |
| *TIP41* | Nö-0 | 26.91 |
| *TIP41* | *pfg1-3* | 26.79 |
| *TIP41* | *pfg1-3* | 27.09 |
| *TIP41* | *pfg1-3* | 26.64 |
| *TIP41* | *pfg1-3 pap1-4* | 26.87 |
| *TIP41* | *pfg1-3 pap1-4* | 26.85 |
| *TIP41* | *pfg1-3 pap1-4* | 26.63 |
| *UBC9* | Col-0 | 23.73 |
| *UBC9* | Col-0 | 23.59 |
| *UBC9* | Col-0 | 23.26 |
| *UBC9* | Nö-0 | 23.04 |
| *UBC9* | Nö-0 | 22.78 |
| *UBC9* | Nö-0 | 22.64 |
| *UBC9* | *pfg1-3* | 22.77 |
| *UBC9* | *pfg1-3* | 22.83 |
| *UBC9* | *pfg1-3* | 22.35 |
| *UBC9* | *pfg1-3 pap1-4* | 22.60 |
| *UBC9* | *pfg1-3 pap1-4* | 22.83 |
| *UBC9* | *pfg1-3 pap1-4* | 22.79 |
