# Supplementary material for: Combined R2R3–MYB transcription factor mutants reveal the regulatory structure of the Arabidopsis thaliana flavonoid biosynthesis pathway
Source: Planta. 2026 Feb 6;263(3):70. doi: 10.1007/s00425-026-04938-8 (PMC12881018; doi:10.1007/s00425-026-04938-8)
Supplement: Supplementary file 2 — Supplementary file2 (DOCX 1061 KB) [file 425_2026_4938_MOESM2_ESM.docx]

**Supplementary Figures**

**Fig. S1** Confirmation of newly generated CRISPR alleles. Shown are parts of the coding sequence and deduced protein sequence of (**A**) *TT2* and (**B**) *PAP3*. Sanger sequencing results of the PCR products, generated using the genotyping primers listed in Table S1, are presented for the *r2r3-myb* mutant alleles. Premature termination codons (PTCs), resulting from the insertions in the mutant alleles are marked with red boxes.

**Fig. S2** Accumulation of proanthocyanidins in the seeds of *A. thaliana* wildtype *and flavonoid multiple r2r3-myb* mutants. The soluble and the insoluble cyanidin fractions are shown. Significant differences between the lines were detected using pairwise independent t‑tests with Bonferroni correction. Significant differences are indicated with brackets and the corresponding adjusted *P*-values. Measurements were performed for six biological replicates (*n* = 6).

**Fig. S3** Accumulation of proanthocyanidins in the seeds of *A. thaliana flavonoid* mutants. The soluble and the insoluble cyanidin fractions are shown. Significant differences between the lines were detected using one-way ANOVA followed by Tukey’s Honestly Significant Difference test. Significant differences are indicated with brackets and the corresponding *P*-values. Measurements were performed for six biological replicates (*n* = 6).

**Fig. S4** Relative anthocyanin accumulation in *A. thaliana* wildtype and flavonoid multiple *r2r3-myb* mutants. **A** In seedlings, grown on MS medium with 0.5 % or 4 % sucrose, anthocyanin accumulation is induced. **B** Rosette leaves of plants grown in high light to induce anthocyanin accumulation. Differences were calculated in R using *rstatix* v0.7.2 and visualized with *ggpubr* v0.6.0. Significant differences between the lines were detected using pairwise independent t‑tests with Bonferroni correction. Significant differences are indicated with brackets and the corresponding adjusted *P*-values. Measurements were performed for six biological replicates (*n* = 6).

**Fig. S5** Gene expression data for *EBGs* (*CHS*, *CHI*, *FLS1*) and *LBGs* (*ANS*, *ANR*) in siliques (**A**) and 3-day-old seedlings (**B**) of *r2r3-myb* mutants and the corresponding wildtype. Bars represent the relative normalized expression (ΔΔCt) to Col-0. Normalization was performed with three reference genes (see Materials and methods). Measurements have been performed for three independent biological replicates each with two technical replicates. Error bars represent the standard error of the mean (SE) of log2 fold changes.

**Fig. S6** Flavonol accumulation in seeds of *A. thaliana flavonoid multiple r2r3-myb* mutants. Thin layer chromatography (TLC) of methanolic extracts of mature, dry seeds. **Left** After staining with diphenylboric acid 2-aminoethylester (DPBA) and polyethylene glycol 4000 (PEG), flavonol glycoside derivatives were identified under UV-light according to Stracke et al. (2007, 2010). Quercetin (Q) derivatives appear orange and kaempferol (K) derivatives appear green; G, glucose; R, rhamnose. **Right** TLC under UV-light, without staining of flavonols. Sinapic acid derivatives are blue. “Faint green”-appearing metabolites, marked with an asterisk (*), are absent in the *chs* mutant, indicating, that they are non-flavonol flavonoids.
